# Stationary and portable sequencing-based approaches for tracing wastewater contamination in urban stormwater systems

**DOI:** 10.1038/s41598-018-29920-7

**Published:** 2018-08-09

**Authors:** Yue O. O. Hu, Nelson Ndegwa, Johannes Alneberg, Sebastian Johansson, Jürg Brendan Logue, Mikael Huss, Max Käller, Joakim Lundeberg, Jens Fagerberg, Anders F. Andersson

**Affiliations:** 1 Science for Life Laboratory, Department of Gene Technology, School of Engineering Sciences in Chemistry, Biotechnology and Health, KTH Royal Institute of Technology, Stockholm, Sweden; 2 Centre for Translational Microbiome Research, Department of Molecular, Tumour and Cell Biology, Karolinska Institutet, Stockholm, Sweden; 3 Department of Medical Epidemiology and Biostatistics, Karolinska Institutet, Stockholm, Sweden; 4 Science for Life Laboratory, Department of Biochemistry and Biophysics, Stockholm University, Stockholm, Sweden; 5 Centre for Ecology and Evolution in Microbial Model Systems, Linnaeus University, Kalmar, Sweden; 6 Stockholm Vatten och Avfall AB, Stockholm, Sweden

## Abstract

Urban sewer systems consist of wastewater and stormwater sewers, of which only wastewater is processed before being discharged. Occasionally, misconnections or damages in the network occur, resulting in untreated wastewater entering natural water bodies via the stormwater system. Cultivation of faecal indicator bacteria (e.g. *Escherichia coli*; *E*. *coli*) is the current standard for tracing wastewater contamination. This method is cheap but has limited specificity and mobility. Here, we compared the *E*. *coli* culturing approach with two sequencing-based methodologies (Illumina MiSeq 16S rRNA gene amplicon sequencing and Oxford Nanopore MinION shotgun metagenomic sequencing), analysing 73 stormwater samples collected in Stockholm. High correlations were obtained between *E*. *coli* culturing counts and frequencies of human gut microbiome amplicon sequences, indicating *E*. *coli* is indeed a good indicator of faecal contamination. However, the amplicon data further holds information on contamination source or alternatively how much time has elapsed since the faecal matter has entered the system. Shotgun metagenomic sequencing on a subset of the samples using a portable real-time sequencer, MinION, correlated well with the amplicon sequencing data. This study demonstrates the use of DNA sequencing to detect human faecal contamination in stormwater systems and the potential of tracing faecal contamination directly in the field.

## Introduction

Many urban areas use separate sewer systems to transport wastewater and stormwater, with the two pipes often buried adjacent to each other. In contrast to wastewater, stormwater is generally discharged into natural water bodies without prior processing in a wastewater treatment plant. Occasional misconnections during initial construction or due to corrosion and damage to the drainage pipes may result in sanitary sewer water (i.e., wastewater) entering the stormwater system and eventually ending up in natural water bodies unprocessed. This is of great concern both from a health and environmental perspective, since wastewater contamination of natural water bodies can bring forth transmission of pathogens and elevated nutrient loads, which in turn may cause eutrophication^1–3^.

For decades, faecal coliforms (e.g., *E*. *coli*) and enterococci have been extensively used as indicators for assessing the contamination level of water samples due to their high prevalence in human faeces, high growth rates, and ease of cultivation^4^. However, they are not in any way perfect because closely related strains, which are hard to distinguish via culturing, exist in the intestines of other animals. Moreover, they can grow on substrates in the environment, and environmental strains exist^5^, which may further lead to false positive results. Another drawback of culture-dependent methods is the temporal aspect: though some culturing-based methods require limited hands-on work, these methods require at least 18 hours of culturing to yield reliable results.

As an alternative to the culture-dependent methods, approaches based on the detection of specific signature molecules are used for tracking anthropogenic wastewater contamination. One example is the detection of caffeine, a molecule exclusive to human waste^6–8^. However, the chemical essays are usually expensive, and the consumption of caffeine varies among human populations. Another example is the detection of specific DNA sequences. Polymerase Chain Reaction (PCR) and quantitative PCR (qPCR) has been extensively applied during the past few decades for detecting genetic material of specific microbial taxa from environmental samples^2,7,9–16^. These methods avoid the problem that many microbes are hard to culture^17^ and allow the detection of human-specific microbial strains such as *E*. *coli* H8 and *Bacteroides* HF183^16,18,19^. As compared to culturing, these methods are faster (e.g., qPCR analysis only takes a few hours) but are more laboratory- and equipment-intensive. And so far, only the Covalently Linked Immunomagnetic Separation/Adenosine Triphosphate (Cov-IMS/ATP) technique can quantify faecal indicator bacteria in the field, though being restricted to the microorganisms *E*. *coli* and *Enterococcus* spp.^20,21^. It is also less sensitive than the culture-dependent methods and of no avail with regard to the detection of host-specific *E*. *coli* or *Enterococcus*. Therefore, exploring new techniques that can track and quantify human pollution in the field is of great interest.

With high-throughput sequencing techniques, it is now possible to obtain detailed profiles of microbial communities in environmental samples rather than just detecting a specific group of microbes. Sequencing of PCR-amplified taxonomic marker genes (i.e., amplicon sequencing, which at times is referred to as ‘metabarcoding’), such as ribosomal RNA genes (rRNA genes), gives a relatively unbiased view of a sample’s taxonomic composition^22–25^. Shotgun metagenomic sequencing provides, in addition to taxonomic composition, also information on functional genes (e.g., antibiotic resistance or toxin genes) and, for well-characterised microbiomes, allows taxonomic profiling at a higher resolution compared to metabarcoding^26^. Although high-throughput sequencing allows assessing different aspects of water quality (e.g., detection of antibiotic resistance)^27^ and identifying suitable indicator groups for different purposes^28,29^, this approach is limited by the high costs and non-portability of instruments. The recent development of a low-cost, cell phone-sized, single-molecule real-time sequencer from Oxford Nanopore Technologies Ltd (Oxford, UK; ONT), however, opens up possibilities for carrying out the sequencing in the field. Its high sequencing error rate has made metagenomic sequencing problematic, but the latest upgrade brought about a drop in its error rate from 38% to approximately 10%, thus rendering this methodology more attractive^30,31^.

In this study, we compared the culture-dependent gold standard, the IDEXX Colilert-18^®^ test, with 16S rRNA gene amplicon sequencing on the Illumina MiSeq platform and shotgun metagenomic sequencing on the portable ONT MinION device in an attempt to assess contamination levels in 73 stormwater samples from the Stockholm city area. The main goals were to (i) evaluate the accuracy of the traditional, culture-dependent method by comparing it with the two sequencing-based methods; (ii) track contamination sources using information from the microbial communities gained by high-throughput sequencing; and (iii) evaluate the feasibility and accuracy of using the portable sequencer to determine wastewater contamination in stormwater systems.

## Results

### Comparison between Illumina MiSeq amplicon sequencing and *E*. *coli* culturing

**Figure 1:**
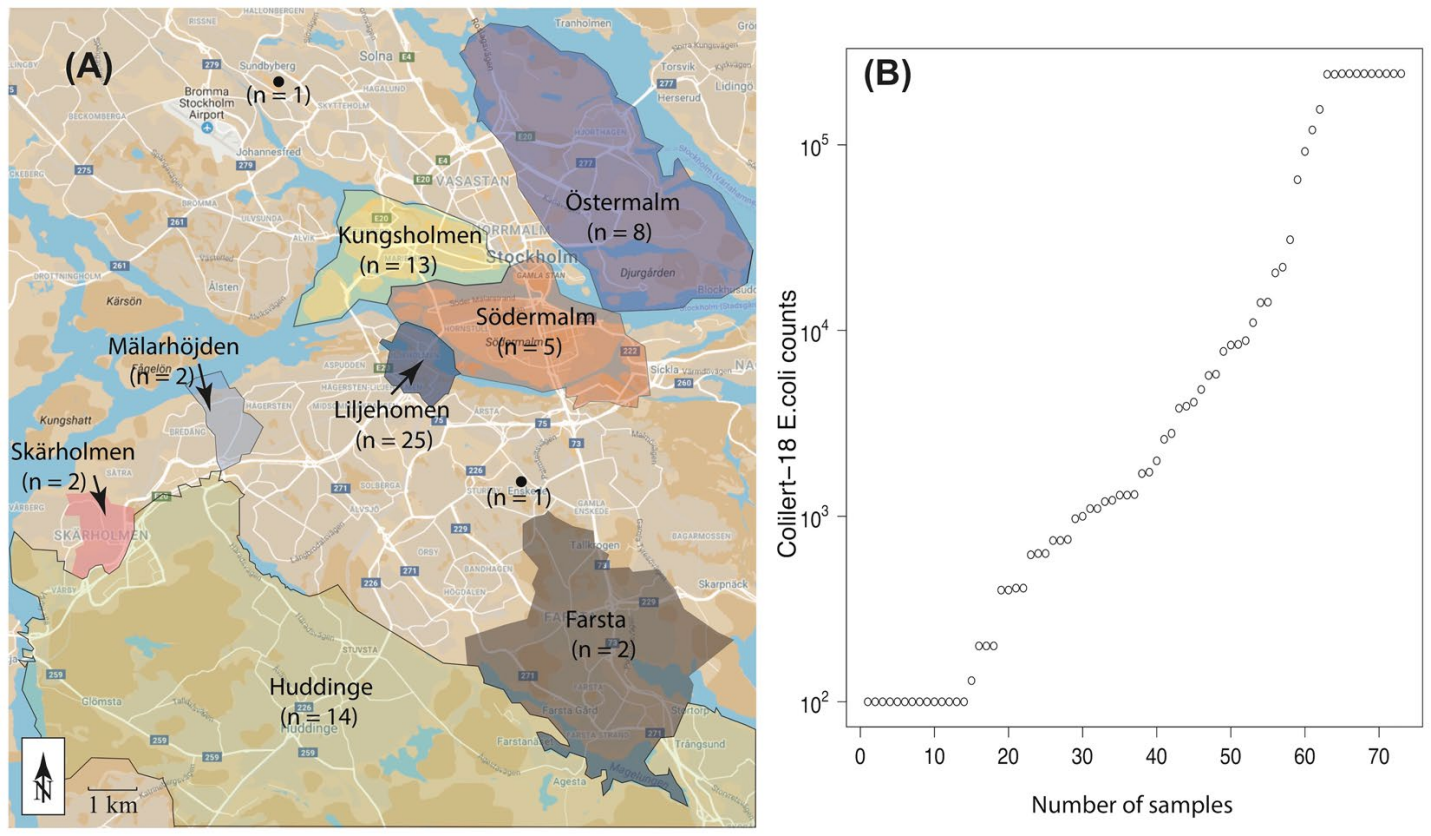
(**A**) Map showing the sampling locations. (**B**) Distribution of *E*. *coli* Most Probable Number (MPN) counts determined by the Colilert^®^-18 system in the 73 stormwater samples. The y-axis is shown in log scale. The map was created manually using Adobe Illustrator CC 2015^63^ by modifying images from Google Maps^64^.

Stormwater samples were collected in duplicate from 73 stormwater manholes distributed around the city of Stockholm (Fig. 1A). A sample’s first field duplicate was used for *E*. *coli* culturing, while its respective second duplicate was subjected to DNA sequencing. An additional water sample was collected from the primary sedimentation tank of a Stockholm wastewater treatment plant (the Bromma wastewater treatment plant; 320,000 population equivalents) to represent a typical wastewater sample.

The *E*. *coli* count data generated with the Colilert^®^-18 test varied from <100 to ≥242,000 most probable number (MPN) per 100 ml of water (note that 242,000 was the upper limit of detection). Fourteen samples had *E*. *coli* counts of <100, eight ≥242,000, and the median was 1,310 (Fig. 1B), indicating that most of the stormwater samples showed low levels of *E*. *coli* contamination (Stockholm Vatten och Avfall AB [i.e., the Stockholm Water Company] usually considers samples with counts ≥8,000 as potentially contaminated).

Amplicon sequencing yielded an average of 14,017 (range: 6,070–30,820) sequencing reads per sample. After correcting for Illumina sequencing errors^32^, an average of 13,807 reads per sample and a total of 20,507 sequence types (unique sequences) were obtained (Supplementary Table 1). The vast majority of the sequence types (20,473) were classified as *Bacteria* at a bootstrap confidence level ≥70%. With the same threshold, 17,345, 16,567, 13,877, and 11,200 sequence types could be classified to at least phylum, class, family, and genus level, respectively. Although the primers used in this study targeted both bacteria and archaea, the sequenced prokaryotes are henceforth referred to as bacteria since only 0.08% of the reads (94 sequence types) were classified as archaea.

**Figure 2:**
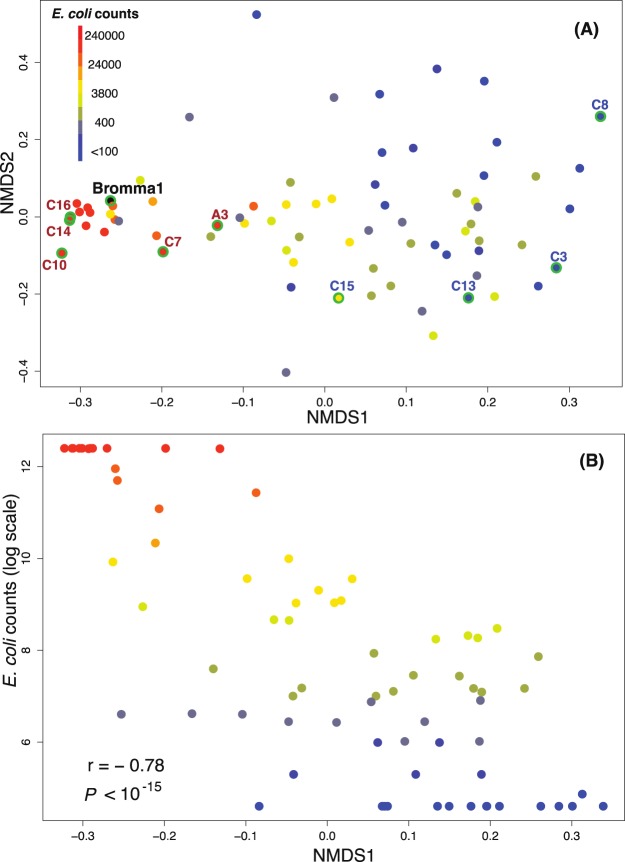
Ordination of samples based on similarity in bacterial community composition and correlation with *E*. *coli* culturing counts. (**A**) Ordination of bacterial communities using NMDS based on Spearman rank order correlation coefficients. Samples are coloured according to their *E*. *coli* counts measured by culturing (Colilert-18^®^) except for the Bromma wastewater treatment plant sample that is coloured in black. Samples with green contours are the ten samples confirmed to be contaminated (red text) or not contaminated (blue text) by wastewater according to follow-up investigations. (**B**) Comparison between community composition (NMDS1) and *E*. *coli* density (*E*. *coli* culturing counts). Spearman rank order correlation coefficient (rho) and p-value are indicated.

In order to investigate how the amplicon sequencing-based overall community composition was correlated with the *E*. *coli* culturing counts, non-metric multidimensional scaling (NMDS) analysis was conducted (Fig. 2). Samples with high *E*. *coli* counts (>200,000) grouped together in the NMDS plot, embedding also the Bromma wastewater treatment plant sample and indicating that samples with high *E*. *coli* counts exhibited similar bacterial community compositions to the wastewater treatment plant sample (Fig. 2A). The samples with lower *E*. *coli* counts were more dispersed in the NMDS plot, indicating that they displayed higher inter-sample variation than the high *E*. *coli* count samples (Fig. 2A). The first axis of the NMDS correlated strongly with the *E*. *coli* culturing counts (Pearson *r* = −0.78, *P* < 10^−15^, Fig. 2B).

**Figure 3:**
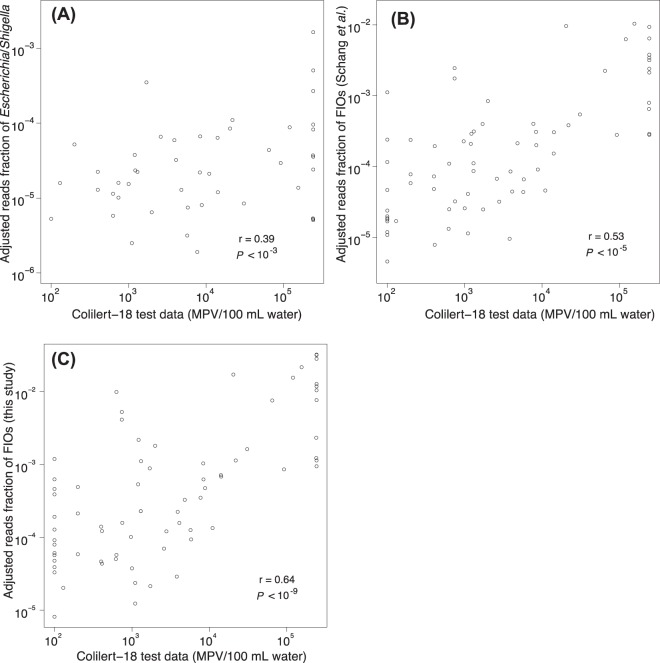
Comparison between *E*. *coli* counts (MPN/100 mL raw water) observed with Colilert-18^®^ test and the adjusted fraction of sequenced amplicon reads annotated as (**A**) the genera *Escherichia/Shigella*. (**B**) FIOs defined by Schang^33^ *et al*., and (**C**) FIOs defined in this study. Both axes are shown in log scale. In (**A**) and (**B**) 26 and 2 samples, respectively, contained 0 reads of the target taxa, and could therefore not be converted to the log-scale and thus not shown in the plots.

To more directly compare results from amplicon sequencing with *E*. *coli* culturing, *E*. *coli* counts recorded for the two methods were correlated against each other. As amplicon sequencing data only reflects relative counts (compared to the absolute abundances in the form of MPN per 100 ml of water for the *E*. *coli* culturing data), the amplicon sequencing data was normalised by means of multiplying the sequence type relative abundances with the amount of DNA extracted per volume of water. Although far from perfect, the amount of extracted DNA per volume of water should serve as a proxy for the total microbial concentration in the sample and, hence, this normalisation should make the abundances of the sequence types more comparable between samples. The normalised fraction of sequencing reads classified as *Escherichia*/*Shigella* (note that these two genera are classified as one group using the RDP classifier) displayed a moderate but significant correlation with the *E*. *coli* culturing counts (Pearson *r* = 0.39, *P* < 0.001; Fig. 3A). Interestingly, this correlation was weaker than between the *E*. *coli* culturing counts and overall community composition (see Fig. 2B). Summing the reads from a list of 20 faecal indicator organisms (FIOs) that was compiled by Schang *et al*.^33^ based on a set of published studies, we, however, obtained a slightly better correlation with the culturing counts (Pearson *r* = 0.53, *P* < 10^−5^; Fig. 3B), which agrees with the results recorded by Schang *et al*.^33^. An alternative approach for defining FIOs is to directly match sequences with human gut microbiome sequences. By BLAST searching our sequence types against data from faecal samples collected from 48 individuals^34^, 1,400 sequence types were identified that displayed ≥99% identity to sequences affiliated with the human gut microbiome. Using this set of sequences, the correlation with the *E*. *coli* culturing counts was further improved (Pearson *r* = 0.64, *P* = 10^−9^) (Fig. 3C).

### The Lake Trekanten area

**Figure 4:**
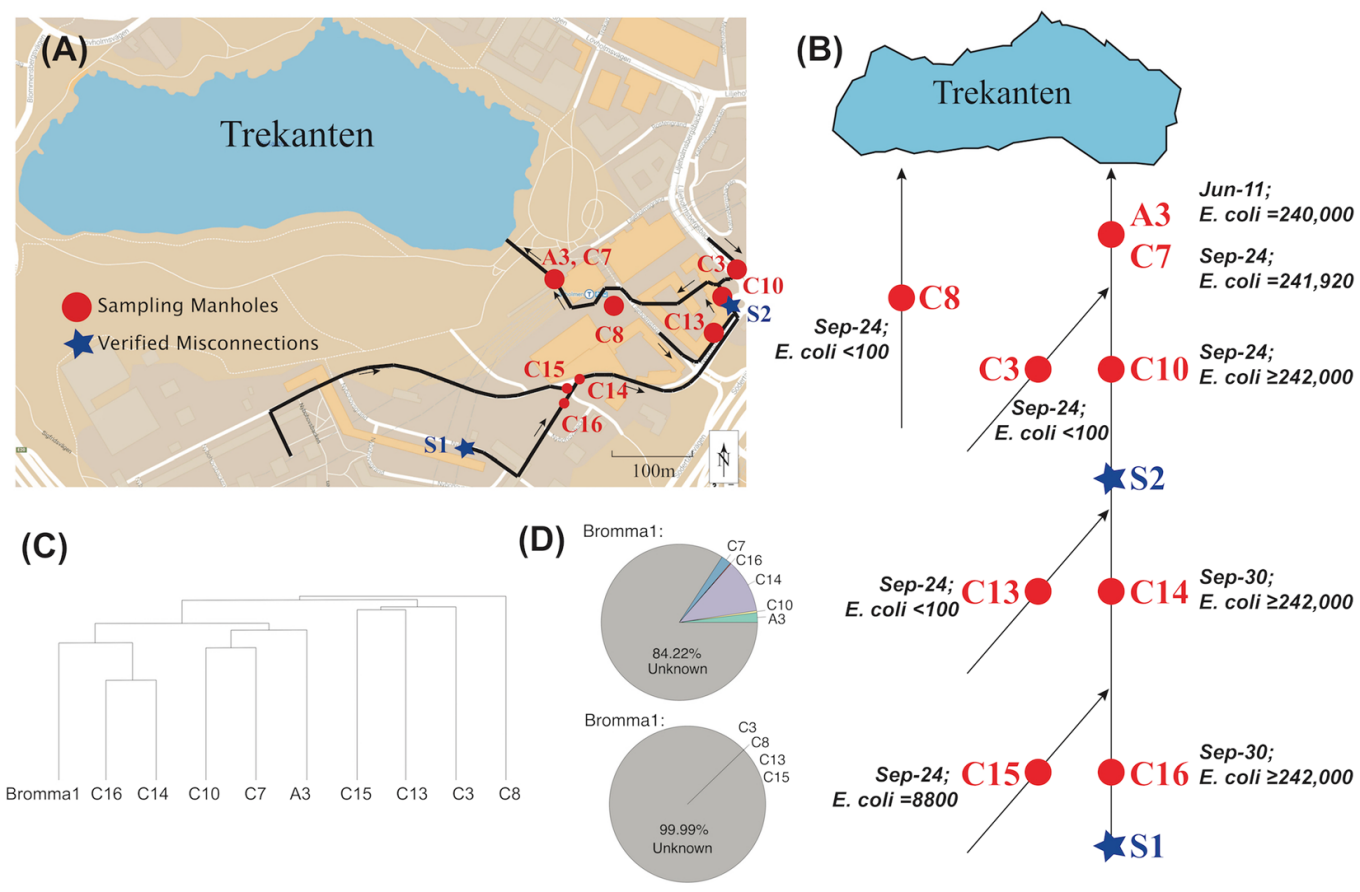
The Lake Trekanten stormwater pipe systems. (**A**) Map showing the Lake Trekanten region. (**B**) Schematic view of the drainage flow in stormwater pipes in that region. Samples were taken from two adjacent but independent stormwater pipe systems near Lake Trekanten. The direction of flow is indicated by arrows. The red dots show the locations of the sampled manholes. The red and black texts indicate a sample’s name and its *E*. *coli* culturing counts, respectively. Samples A3 and C7 were taken from the same manhole on two different sampling occasions. The blue stars present the two locations, where misconnections were detected, with sanitary sewer pipes being connected to the stormwater pipes. Source 1 contained wastewater from toilets and bathrooms, while Source 2 consisted of wastewater from kitchens, toilets, and bathrooms. (**C**) Results from hierarchical clustering analysis based on Bray-Curtis similarities of sequence read abundances. Sample names and sampling dates are indicated. The maps used in (**A**) and (**B**) were created manually using Adobe Illustrator CC 2015^63^ by modifying an image from Google Maps^65^. (**D**) Results of SourceTracker^40^ analysis, showing the contribution from the wastewater-contaminated (upper pie chart) and -uncontaminated (lower pie chart) stormwater samples to the bacterial community of the wastewater treatment plant sample.

One of the areas that was sampled is a municipal community adjacent to Lake Trekanten in Liljeholmen (Fig. 4A); a small lake that has suffered from severe eutrophication during recent years^35^. Based on the *E*. *coli* culturing counts, misconnections or damages in the stormwater system leading into Lake Trekanten were suspected, whereupon, in 2014, after the sequencing data had been generated, Stockholm Vatten och Avfall AB carried out a follow-up investigation in that area. And indeed, two misconnections could be identified with wastewater from two different sources being connected to the stormwater system (Fig. 4B). Nine of the 73 examined stormwater samples have been collected from that region, allowing a comparison of bacterial community composition between contaminated and non-contaminated samples within the same area.

Clustering the samples based on bacterial community composition resulted in two major clusters, one cluster consisting of contaminated samples, that is samples downstream of the two misconnections, and one cluster consisting of non-contaminated samples (Fig. 4C). The Bromma wastewater treatment plant sample clustered together with the contaminated samples. Notably, the contaminated samples formed two subclusters congruent with the manholes’ locations downstream of the two different contamination sources. Finally, one manhole was sampled on two occasions (June and September), with its two samples ending up in the same subcluster.

**Figure 5:**
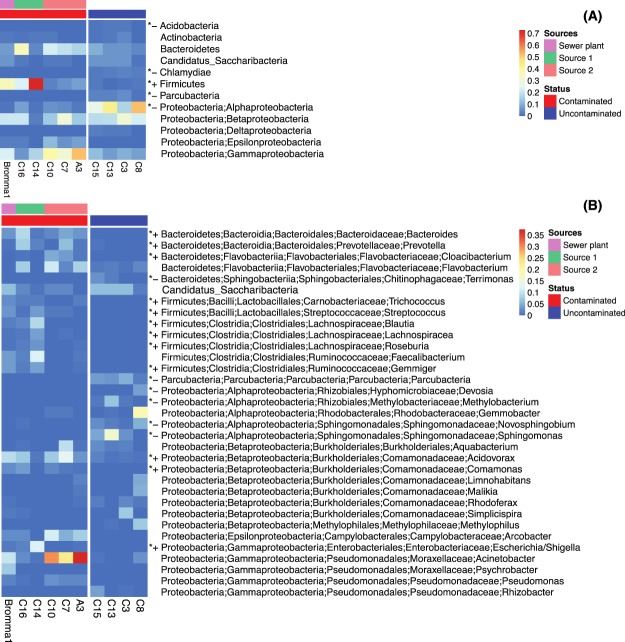
Bacterial community composition in samples from the Trekanten area and the Bromma wastewater treatment plant. The samples were divided into two groups (wastewater contaminated or uncontaminated). Panel (**A**) shows phyla/classes (phylum *Proteobacteria* is divided into classes) with mean relative abundance >10^−3^ in at least one of the groups, while panel (**B**) depicts genera with mean relative abundance >10^−2^ in at least one of the groups. Taxa displaying significant difference in relative abundance between the two groups are marked with an asterisk (Wilcoxon rank sum test, False Discovery Rate-adjusted *P* < 0.05), and higher and lower relative abundances in the contaminated compared to the uncontaminated group are shown as “+” and “−”, respectively.

Figure 5 illustrates the bacterial composition of the Trekanten samples. Unsurprisingly, typical human gut microbiome taxa displayed significantly higher relative abundances in the samples downstream of the misconnections, while aerobic bacteria were more abundant in the uncontaminated samples. At the phylum/class level, *Firmicutes*, a major human gut phylum^36^, displayed >20 times higher relative abundances in the contaminated compared to the uncontaminated Trekanten samples, while *Alphaproteobacteria*, a class comprising mainly aerobic microbes, were nearly ten times more abundant in the uncontaminated compared to the contaminated samples (Fig. 5A). At the genus level, besides many *Firmicutes* and three *Bacteroidetes* genera (*Bacteroides*, *Prevotella*, and *Cloacibacterium*), two genera from the *Betaproteobacteria* (*Acidovorax* and *Comamonas*), and the classical faecal indicator *Escherichia/Shigella* (*Gammaproteobacteria*) also showed significantly higher abundances in the contaminated samples (Fig. 5B). *Bifidobacterium* (phylum *Actinobacteria*), another common human intestinal microbe, was also significantly more abundant in the contaminated group (not shown in Fig. 5 due to its low abundance).

The overhaul of the Trekanten drainage system revealed that the two sources of wastewater that had been wrongly connected to the stormwater pipes were of different character. “Source 1” (Fig. 4B) comprised wastewater originating exclusively from toilets and bathrooms from a temporary building, while “Source 2” contained wastewater draining toilets, bathrooms, laundry, and kitchens of a housing complex made up of 85 apartments and offices. Interestingly, microbial communities sampled downstream of the two pollution sources demonstrated different features (Fig. 5). Samples downstream of “Source 2” had lower relative abundances of all *Firmicutes* genera but 20 times higher relative abundance of *Acinetobacter* from *Gammaproteobacteria* (29.9% on average). Among all Trekanten samples, 106 sequence types were classified as *Acinetobacter* of which 96 were recorded in the three samples downstream of “Source 2”. *Acinetobacter* was also well represented in the Bromma wastewater treatment plant sample (10.2% of the bacterial community), corroborating earlier studies^37–39^.

To verify that the samples downstream of the two misconnections demonstrated signatures of wastewater contamination, we used SourceTracker analysis^40^. Here, the stormwater samples acted as sources, while the wastewater treatment plant sample was included as the sink. The contaminated sources explained 15.78% of the microbial community found in the wastewater treatment plant, while the non-contaminated sources explained <0.01% (Fig. 4D). Although stormwater from these sites in reality do not reach the treatment plant, this analysis demonstrates that the contaminated sites display signatures of wastewater contamination.

### Comparison between MinION shotgun sequencing and Illumina MiSeq amplicon sequencing

**Figure 6:**
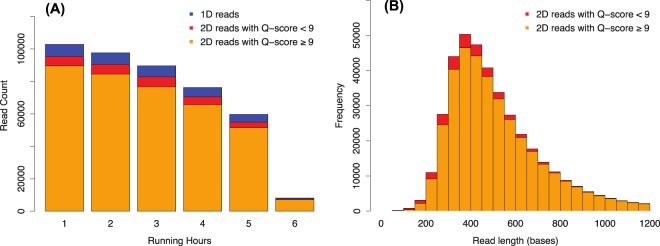
(**A**) Read yields (number of sequencing reads per hour) and their quality status during the first six hours of MinION shotgun metagenomic sequencing. The blue color indicates the count of 1D reads, the red and orange colors indicate the counts of 2D reads with either a mean Q-score of < or ≥9. (**B**) Read length distribution of 2D reads. Only the 2D reads within the length range of 0 to 1,200 bases were shown, including 96.6% of the total amount of 2D reads. The longest 2D read is 34,840 bases long, and the longest 2D read that passed the quality filter (mean Q-score ≥9) is 6,670 bases long.

Five samples with either high or low *E*. *coli* culturing counts (≥242,000 or <100 MPN per 100 ml of water) were subjected to MinION shotgun sequencing. After six hours of sequencing, 434,262 sequencing reads with an average length of 602 base pairs (bp) were obtained (Fig. 6). After quality filtering, 375,111 barcoded 2D-reads (reads sequenced from both directions) with an average Q-score of 13.2 (equivalent to an expected sequencing error rate of ~5%) were used for the downstream analysis.

**Figure 7:**
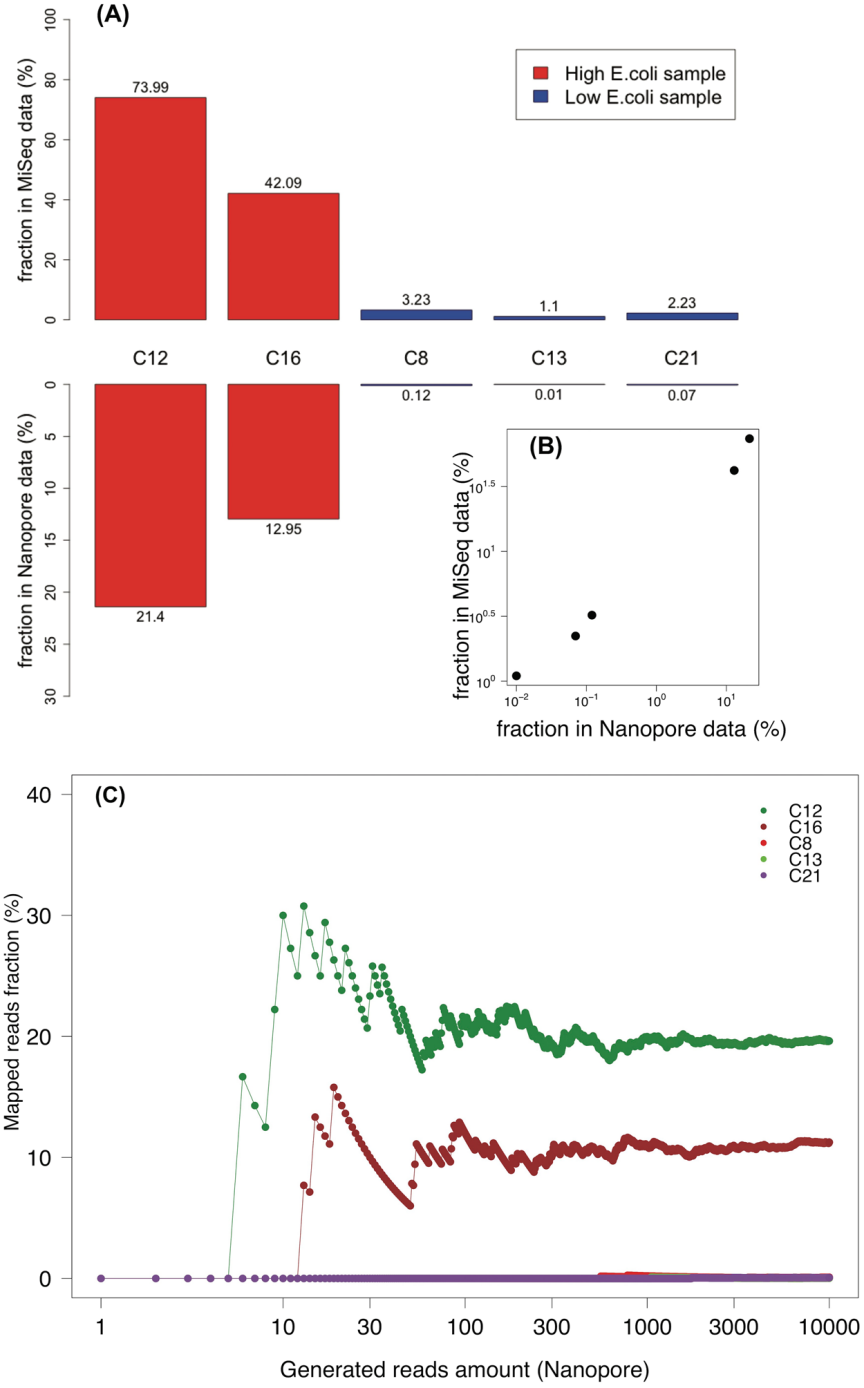
Fraction of reads mapping to human gut microbiome sequences for the five samples. (**A**,**B**) Comparison of the mapping ratio between sequences stemming from amplicon and shotgun metagenomic sequencing, respectively. Samples represented by red and blue bars have *E*. *coli* culturing counts >242,000 and <100 MPN, respectively. The reads fraction was calculated based on 10,000 reads subsampled from each sample for either approach. (**C**) The mapping ratio of Nanopore MinION data calculated at different numbers of reads mapped. The x-axis is shown in log scale. The values of C8, C13 and C21 are generally 0, which is why their curves are overlapping.

In order to quantify the faecal contamination of the samples, shotgun reads from each sample were mapped to a comprehensive human gut microbiota gene dataset, comprising 9.9 million gene sequences^41,42^. Reads were trimmed to a length of 400 bp to minimise biases due to read length differences (although the read length distributions of the five samples were rather similar; data not shown). 10,000 trimmed reads were randomly subsampled from each sample and matched to the human gut microbial genes, using identity and alignment length thresholds of 90% and 200 bp, respectively. We used a 90% identity threshold to roughly match the sequences at the species level (intraspecies identity of orthologous genes is usually >94%^43^), while allowing for 5% sequencing errors. The proportion of reads that matched ranged from 0.01% to 21.04% and these numbers correlated well with the proportion of amplicon reads matching FIOs (as defined in this study) for the same samples (Fig. 7A,B). We also assessed how many reads were necessary to reliably estimate contamination levels of these samples. For all of the five samples analysed, at 1,000 reads contamination estimates had stabilised, and differed by <1% from the estimates obtained from 10,000 reads (Fig. 7C). This corresponds to only six minutes of sequencing given five samples are to be sequenced in parallel.

## Discussion

In this study, we have compared the performance of the classical *E*. *coli* culturing method with two DNA sequencing-based approaches for tracking wastewater contamination in urban stormwater systems. Overall, the two sequencing-based methodologies showed similar trends to the results obtained from the conventional culturing-based method: that is the proportion of sequencing reads mapping to human gut microbiome sequences significantly correlated with the *E*. *coli* culturing counts. Although concerns have been raised with respect to using *E*. *coli* as a wastewater indicator because *E*. *coli* is not exclusive to humans and because of its high survival capacity in the environment^33,44,45^, the findings made in this study indicate that it still can be a useful marker for faecal contamination. Interestingly, *E*. *coli* culturing counts correlated stronger with the proportion of amplicon sequencing reads matching human microbiome sequences than to the proportion of reads classified as *Escherichia/Shigella*. This is unlikely an effect of mismatches between the employed PCR primers and *E*. *coli* sequences (93.7% of *E*. *coli* sequences in RDP matched perfectly to the primer pair used). It is more likely an effect of the small number of reads that are classified as *Escherichia/Shigella*, which make the relative abundance estimates noisy; using a larger number of indicator sequence types gives more robust estimates (y-axes scales differ between the different panels of Fig. 3).

For the five samples that were also analysed through shotgun metagenomic sequencing, a high correlation with the *E*. *coli* culturing counts as well as amplicon sequencing data was observed. This suggests that, despite the relatively high sequencing error rate, shotgun metagenomic sequencing on a MinION device can adequately assess the status of faecal contamination in environmental samples. The lower proportion of sequencing reads matching human microbiome sequences observed for the shotgun metagenomic sequencing approach compared to that of the amplicon sequencing method is probably due to a combination of reasons. First, some shotgun metagenomic reads may stem from mainly intergenic regions and will, as such, not be matched to reference sequences from the database. Second, sequences may well be associated with the human gut microbiome but because the reference database is incomplete show up as no match. Third, sequencing errors, prone to MinION sequencing, may cause identity levels of matches drop below the cutoff level. Finally, it could also potentially be due to a background of environmental eukaryotic DNA that is not captured during the 16S amplicon sequencing but only in the shotgun sequencing. The fact that the ratio between the amplicon and shotgun match rates increases (from 3.5 to 31) indicates a background of false positives in the amplicon data (i.e. that a subset of our FIOs also exists in uncontaminated stormwater).

Specificity is an important issue when screening water for signs of pollution or contamination. Using traditional faecal indicator bacteria may give rise to false-positives as pet, rodent, or bird faeces, yet animal faeces in general, also contain such bacteria^44^. DNA sequencing has the potential to not only estimate levels of contamination but also determine the source of it. As extensive intestinal microbiome datasets of different animals emerge, it will be possible to determine animal sources with greater precision and confidence^16^. In this study, the bacterial communities of the wastewater contaminated samples from the Lake Trekanten area clustered according to contamination source. This corroborates earlier studies that found that the content of wastewater can reflect lifestyle and diet of the population^46,47^. For example, the high levels of *Acinetobacter* in the samples downstream of one of the sources could reflect an abundance of this microbe in the source (this genus has been found in high levels in, for instance, kitchen sponges^48^). Alternatively, since *Acinetobacter* is known to thrive in wastewater treatment plants^37,39,49,50^, it is possible that this aerobic bacterium enriched in the wastewater-contaminated stormwater on its way to the sampling points. This is also a very interesting scenario, since it implies that the microbial community carries information on how much time has elapsed since the faecal matter entered the aerobic conditions of the water system. This hypothesis can be tested in an experimental setting.

Another issue in this regard is sensitivity. With the traditional culture-dependent methods, detection limits are in theory as little as one viable indicator cell per volume analysed. Typically for the Colilert-18^®^ test, 100 ml of water are analysed. When performing broad-taxonomic range amplicon or shotgun metagenomic sequencing, the sequencing reads of indicator bacteria will be diluted with reads from other bacteria sequenced as part of the library. In this case, the detection limit will depend on the ratio between FIO bacteria and other bacteria in the community. From the set of stormwater samples that had <100 MPN, on average 1.15 ng DNA/ml of water (range 0.16–3.73 ng/ml) was extracted. If all this DNA represented bacteria with an average genome size of 3 Mbp, it would correspond to 355,693 genomes/ml of water. This is roughly on par with reports in the literature of stormwater containing between 10^2^ and 10^6^ bacterial cells per ml of water^51–53^. For obtaining a single read from an FIO present as a single cell in this sample would require on average 10^2^–10^6^ sequencing reads, which is achievable with current sequencing technologies, and thus a sensitivity comparable to that of selective culturing can be obtained. An advantage of culture-dependent methods is that they yield absolute counts, while sequencing data is only relative. However, by adding a DNA standard before DNA extraction, absolute quantifications can be achieved^54^ (the spike-in DNA can moreover serve as an estimation of sequencing error rates).

**Figure 8:**
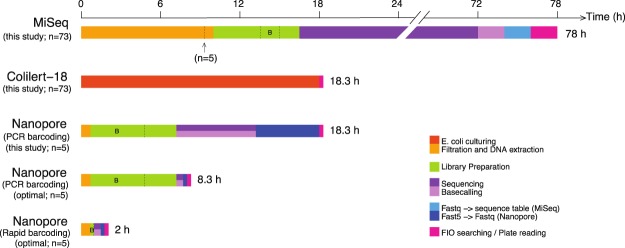
Expenditures of time for assessing levels of human faecal contamination in water samples with different approaches. The first three bars demonstrate the amount of time needed with the experimental settings applied in this study, while the last two bars show the feasible time requirements of the Nanopore-based approach with settings optimised based on the results of this study. Computing time for sequence and data analysis (i.e., the steps after the actual sequencing) was calculated based on the computing power of 16 threads. The arrow under the MiSeq timeline indicates the starting point if only five samples had been processed. Time for the Colilert-18^®^ test would not change regardless of sample size. The time for the barcoding procedure in the Nanopore library preparation was segmented from the library preparation hours and marked with “B”. The base-calling procedure for Nanopore can be conducted while the sequencing is ongoing. The optimised Nanopore timelines (the 4th and 5th bars) demonstrate the feasible time usage for assessing five water samples by using different barcoding approaches (ligation-based or transposase-based) with the sequencing depth of 1,000 reads per sample.

In addition to being sensitive and specific, an ideal monitoring tool should be quick and cheap. Figure 8 illustrates the expenditures of time for the three methods used in this study. The MiSeq amplicon procedure requires more than three days for it to complete, while both the Colilert-18^®^ test and MinION shotgun sequencing can be finished within 24 hours. With regard to amplicon sequencing, sequencing time may be shortened by 24 h if using the Illumina MiSeq *V2 300-cycle* reagent kit. This comes, however, at the expense of read lengths. Yet, the 2 × 150 bp long reads should still provide sufficient taxonomic resolution. As for shotgun metagenomic sequencing, the sequencing itself ran for six hours, though our results indicated that six minutes would have sufficed (corresponding to 1,000 reads per sample). Here, the time needed will, however, have to be increased linearly with the number of samples, and greater sequencing depth should be aimed at to detect low levels of contamination. Downstream FASTQ conversion and FIO searching can be done in 40 minutes. With a newly released barcoding kit that conducts DNA fragmentation and adapter ligation simultaneously, library preparation can be achieved within 10 minutes, shortening the time from filtering to results to two hours. Pricewise, the Colilert-18® test is the cheapest ($32 per sample). MiSeq amplicon sequencing is more cost efficient than MinION shotgun metagenomic sequencing in terms of price per gigabase ($96 vs. $515). With a new multiplexing kit from Nanopore it is now possible to run 96 samples in parallel on the MinION device, resulting in library preparation plus sequencing costs of $55 per sample. Running the same number of samples on the Illumina MiSeq platform would amount to $84.

From the set of methodologies compared in this study, only the portable Oxford Nanopore MinION device shows promise of carrying out the full range of steps involved in the detection of contamination in the field itself. However, to be able to actually conduct metagenomic sequencing on the MinIOn device in the field, a number of steps would need to be adapted to the situation in the field. The Rapid Low Input Nanopore kit requires only 10 ng of genomic DNA, which allows collecting microbes from only 10–20 ml water. Filtration could then be performed in the field by using a syringe filter (instead of using a pump-driven system with filtration manifolds). However, DNA extraction usually requires vortexing and centrifugation, which makes this the bottleneck step. Thus, there is a high demand for portable systems for DNA extraction. Another possible bottleneck may be the bioinformatic analyses that require high computing power. Here, we adopted an extensive (9 million genes) human gut flora database (integrated gene catalog (IGC) database of human gut microbiome sequences^42^) to distinguish human gut bacteria from background bacteria in stormwater. Improving the bioinformatic strategy by possibly utilising a smaller reference database of core genes, that would allow for a more rapid analysis on a normal laptop, would be desirable.

## Methods

### Sampling

Stormwater samples were collected from 73 manholes distributed around Stockholm city on six occasions from June - September 2013 in collaboration with the Stockholm Water Company (Stockholm Vatten och Avfall AB; Stockholm, Sweden). Sampling dates and locations are listed in Supplementary Table 2. At each sampling site, 100 ml and 1 l of raw stormwater were collected in a sterile 100 ml glass bottle and sterile polycarbonate carboy to run the *E*. *coli* culturing assays and high-throughput sequencing analyses, respectively. The field duplicates were transported cooled on ice to the laboratory, where culturing and filtration was conducted on the very same day. An additional 1 l of raw sewage water was collected in a sterile polycarbonate carboy from the primary sedimentation tank of the Stockholm Bromma wastewater treatment plant on June 10, 2013, and transported cooled to the laboratory for filtration the same day.

### *E*. *coli* culturing assay

The IDEXX Colilert-18^®^ test (IDEXX Laboratories Inc.; Westbrook, ME, USA) was performed on the stormwater samples to quantify viable *E*. *coli* in each sample, following the instructions given by the manufacturer. The Colilert-18^®^ method is approved and included in European Standard Method (EN ISO 9308-2) and U.S. Environmental Protection Agency Standard Methods for the enumeration of coliform bacteria and *E*. *coli* in water^55,56^. The assay was carried out by a commercial laboratory in Stockholm (Eurofins Environment Testing Sweden AB; Stockholm, Sweden). In brief, the sample is hereby divided into a number of wells and - based on the number of wells in which *E*. *coli* growth was detected using a fluorogenic reaction - a Most Probable Number (MPN) of *E*. *coli* cells in the sample is calculated applying a statistical model.

### DNA extraction

Subsamples of 0.5 to 1 l from the respective field duplicate and the wastewater treatment plant sample were filtered through sterile Water Filter Units (MO BIO Laboratories Inc.; Carlsbad, CA, USA), collecting microbes onto a 0.22 μm pore-size Polyethersulfone membrane. Filters were kept frozen (−20 °C) overnight, and DNA extraction was conducted the next day. The PowerWater^®^ DNA Isolation kit (MO BIO Laboratories Inc.) was used for genomic DNA extraction, following the manufacturer’s instructions. The extracted DNA was subsequently quantified by a Qubit^®^ 2.0 Fluorometer (Qubit-IT^TM^ dsDNA HS Assay kit; Invitrogen; Carlsbad, CA, USA) and stored at −20 °C until further analysis.

### Illumina MiSeq library preparation and sequencing

The 73 stormwater and wastewater treatment plant samples were subjected to 16S rRNA gene amplicon sequencing on the Illumina MiSeq platform (Illumina Inc.; San Diego, CA, USA). The sequencing library was prepared according to a two-step PCR procedure. The 1^st^ PCR (25 cycles) step amplified the hypervariable V3–V4 region of the prokaryotic 16S rRNA gene, while the 2^nd^ PCR (10 cycles) step attached dual indexes to both ends of the 16S amplicons in order to barcode each sample individually. The 16S primers used in the 1st PCR step were primers 341′F (CCTAHGGGRBGCAGCAG)^25^ and 805R (GACTACHVGGGTATCTAATCC)^57^ both of which had been modified by means of extending their 5′-ends with Illumina adapter sequences to enable the actual barcoding of samples. Once amplified, the 16S amplicons were purified with 8.8% Polyethylene Glycol 6000 precipitation buffer (Merck Millipore; Billerica, MA, USA) and CA beads (Dynabeads^®^ MyOne^TM^ Carboxylic Acid, carboxylic acid-coated superparamagnetic beads; Invitrogen)^58^. The barcoding primers comprise both index sequence and Illumina sequencing handle sequence; the later attaches the amplicons onto the Illumina flow cell to initiate sequencing. In both PCR steps, the KAPA HiFi HotStart ReadyMix (2X; KAPA Biosystems; Wilmington, MA, USA) was used and PCR mixtures were each time prepared according to the manufacturer’s instructions (KAPA Biosystems). Amplicon fragment size and quantification were checked using the DNA 1000 LabChip kit (Agilent Technologies; Santa Clara, CA, USA) on an Agilent 2100 Bioanalyzer and the Qubit-ITTM dsDNA HS Assay kit (Invitrogen) on a Quibit^®^ 2.0 Fluorometer. Finally, after repeating the purification procedure on the now barcoded amplicons, equimolar amounts of samples were mixed and the final amplicon sequenced on an Illumina MiSeq platform (Illumina Inc.) at NGI/SciLifeLab Stockholm using the *V3 600-cycle* reagent kit.

### Oxford Nanopore library preparation and sequencing

Five stormwater samples were shotgun sequenced with the MinION device. These samples were randomly selected from the stormwater samples with *E*. *coli* counts and MPNs culturing counts either >242,000 or <100 MPN per 100 ml, respectively. Approximately 1,200 ng of the genomic DNA of each sample were sheared in microTUBEs (ATA™ Fiber Crimp - Cap 6 × 16 mm; Covaris Inc.; Woburn, MA, USA) with Covaris S2 instrument (Covaris Inc.) to 550 bp-long fragments. The sheared DNA was purified with the QIAquick^®^ PCR purification kit (Qiagen Inc.; Hilden, Germany) before conducting the Nanopore library preparation. The purified genomic DNA fragments (510~840 bp for each purified sample) were PCR-barcoded using the MinION PCR barcoding kit DEV-MAP004 (Oxford Nanopore Technologies; Oxford, UK). The barcoded products were further processed as sequencing library by using the Nanopore Sequencing kit SQK-NSK007 (version R9; Oxford Nanopore Technologies). The procedure of PCR barcoding and library preparation followed the Nanopore archived protocol, PCR barcoding genomic DNA (R9 and SQK-NSK007). After priming the SpotON Flow Cell (FLO-MIN106 R9.4 SpotOn; Oxford Nanopore Technologies) installed on the Oxford Nanopore MinION^TM^ Mk1 B sequencer (Oxford Nanopore Technologies), 75.0 ul of the library were loaded onto the sample port. A 48-h sequencing protocol (NC_48Hr_Sequencing_Run_Flo_MIN106_SQK_LSK208.py) was initiated on the MinKNOW control software (version 1.3.25) to start the sequencing, whereas a 2D Base-calling plus barcoding program (for FLO-MIN106: “2D Base-calling plus Barcoding for FLO-MIN106 250 bp”) was launched on the Metrichor software (version 1.125) to obtain the base-called and demultiplexed fast5 files while the sequencing was ongoing.

### MiSeq sequences analysis

The sequence table (Supplementary Table 1) was built following the DADA2 pipeline^32^ (http://benjjneb.github.io/dada2/tutorial.html). In brief, after checking the quality profiles of the forward and reverse reads, the degenerated primer region (22 bp and 21 bp from the 5′-ends) as well as low-quality tails (15 bp and 70 bp from the 3′-ends) were trimmed from the forward and reverse reads, and read-pairs containing the base “N” or having quality scores below 10 were discarded. Dereplication, error model learning, and sample inference was conducted on the filtered and trimmed reads with DADA2 using default settings. The denoised reads were merged using a minimum of 30 bp overlap tolerating only one mismatch. Chimeric and PhiX sequence variants were removed again with DADA2, and the remaining sequence variants were finally classified with the Ribosomal Database Project (RDP) classifier^59^ (RDP 16S rRNA training set 14, bootstrap value ≥70%).

### Nanopore sequences analysis

The base-called fast5 files that had passed the quality filtering (i.e., barcoded 2D reads with a quality score ≥9) were converted to FASTA format by using the FASTA extraction function in Poretools (version 0.5.1)^60^. Usearch local alignment (Usearch 64-bit, v8.1.1861)^41^ was employed to match the sequences that were trimmed at 400 bp against an integrated gene catalog (IGC) database of human gut microbiome sequences^42^. The alignment search was running with 16 threads and only retrieving the best hits with identities ≥90% and E-values ≥10^−6^ to accelerate the procedure. Alignment results were further filtered such that only hits with a minimum 200-bp alignment lengths were included. The identity cut-off was chosen based on intraspecies average nucleotide identity (around 94%)^43^ and average error rate of the filtered Nanopore sequences (5%; corresponding to the average Q-score of 13.2) (i.e., 90% ≈ 94% × (100–5%)).

### SourceTracker analysis

SourceTracker^40^ was used to verify that Trekanten samples taken downstream of the two misconnections (i.e., contaminated samples) contained a greater proportion of sequences found in the wastewater treatment plant compared to the non-contaminated samples. Thus, two independent SourceTracker analyses were conducted, adopting default settings for each of the two analyses (rarefaction depth = 9,648, alpha = 0.001). In both analyses, the sample taken from the wastewater treatment plant served as the sink, while the Trekanten samples (i.e., contaminated or non-contaminated) were treated as sources.

### Statistical analysis

All statistical analyses and plotting were conducted in R (www.r-project.org) using the R libraries vegan (α-diversity; β-diversity; subsampling; NMDS)^61^, cluster (hierarchical clustering)^62^, and SourceTracker^40^ (v1.0.1)

### Data availability

The sequencing data (both Illumina MiSeq and Oxford Nanopore MinION sequencing data) have been submitted to the European Nucleotide Archive (ENA) repository, under the accession number PRJEB20562. The detailed Illumina MiSeq amplicon library preparation protocol is archived on https://github.com/EnvGen/LabProtocols/.

## Electronic supplementary material

Dataset1

Dataset2
